# Shorter P1m Response in Children with Autism Spectrum Disorder without Intellectual Disabilities

**DOI:** 10.3390/ijms22052611

**Published:** 2021-03-05

**Authors:** Yuko Yoshimura, Takashi Ikeda, Chiaki Hasegawa, Kyung-Min An, Sanae Tanaka, Ken Yaoi, Sumie Iwasaki, Daisuke N. Saito, Hirokazu Kumazaki, Hirotoshi Hiraishi, Mitsuru Kikuchi

**Affiliations:** 1 Institute of Human and Social Sciences, Kanazawa University, Kanazawa 920-1192, Japan; 2 Research Center for Child Mental Development, Kanazawa University, Kanazawa 920-8640, Japan; tikeda@med.kanazawa-u.ac.jp (T.I.); hasegawachiaki1014@gmail.com (C.H.); eirene.akmin@gmail.com (K.-M.A.); tanakast@staff.kanazawa-u.ac.jp (S.T.); kyaoi@med.kanazawa-u.ac.jp (K.Y.); s.iwasaki623@gmail.com (S.I.); mitsuruk@med.kanazawa-u.ac.jp (M.K.); 3 Higher Brain Function & Autism Research, United Graduate School of Child Development, Kanazawa University, Kanazawa 920-8640, Japan; 4 Human Communication Science & Intervention, United Graduate School of Child Development, Kanazawa University, Kanazawa 920-8640, Japan; 5 Faculty of Psychology, Yasuda Women’s University, Hiroshima 731-0153, Japan; saito-d@yasuda-u.ac.jp; 6 Department of Preventive Intervention for Psychiatric Disorders, National Center of Neurology and Psychiatry, Tokyo 187-8553, Japan; kumazaki@tiara.ocn.ne.jp; 7 Department of Biofunctional Imaging, Hamamatsu University School of Medicine, Hamamatsu 431-3192, Japan; hirotoshisakuma@gmail.com; 8 Socio-Neuro Science, United Graduate School of Child Development, Kanazawa University, Kanazawa 920-8640, Japan; 9 Department of Psychiatry and Neurobiology, Graduate School of Medical Science, Kanazawa University, Kanazawa 920-8641, Japan

**Keywords:** autism spectrum disorder, auditory cortex, P1m response, magnetoencephalography

## Abstract

(1) Background: Atypical auditory perception has been reported in individuals with autism spectrum disorder (ASD). Altered auditory evoked brain responses are also associated with childhood ASD. They are likely to be associated with atypical brain maturation. (2) Methods: This study examined children aged 5–8 years old: 29 with ASD but no intellectual disability and 46 age-matched typically developed (TD) control participants. Using magnetoencephalography (MEG) data obtained while participants listened passively to sinusoidal pure tones, bilateral auditory cortical response (P1m) was examined. (3) Results: Significantly shorter P1m latency in the left hemisphere was found for children with ASD without intellectual disabilities than for children with TD. Significant correlation between P1m latency and language conceptual ability was found in children with ASD, but not in children with TD. (4) Conclusions: These findings demonstrated atypical brain maturation in the auditory processing area in children with ASD without intellectual disability. Findings also suggest that ASD has a common neural basis for pure-tone sound processing and language development. Development of brain networks involved in language concepts in early childhood ASD might differ from that in children with TD.

## 1. Introduction

Autism spectrum disorder (ASD) comprises neurodevelopmental disorders characterized by deficits in social communication and restrictive and repetitive patterns of behavior, interests, and activities [1]. Behavioral characteristics peculiar to ASD appear from early childhood [2]. Brain imaging studies have revealed structural and functional features underlying those traits [3,4,5,6,7]. However, because the characteristics of ASD are regarded as continuous variables rather than categorical variables [1] and because of the variety of its symptoms, early brain function and maturation remain unclear. Elucidating the characteristics of atypical brain function in children with ASD is expected to promote an objective understanding of ASD pathology. Furthermore, clarifying the neurological bases involved in language development of children with ASD is expected to contribute to the selection of suitable intervention methods for individuals with ASD who show various language symptoms and is expected to contribute to verifying their effects. Nevertheless, much remains unknown about brain function characteristics in children with ASD and those characteristics’ association with language development.

**Figure 1:**
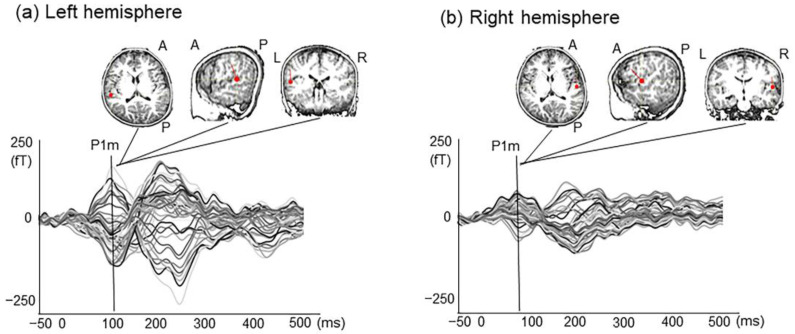
Source modeling of P1m responses evoked by tone stimuli from a 5-year-old male with TD. AEF waveform and dipole location of P1m response in both Heschel’s gyri in a child. The P1m response showed an activity peak at 70–150 ms in the left hemisphere (**a**) and the right hemisphere (**b**). Red arrows indicate the directions of equivalent current dipole orientation. A, anterior; P, posterior; L, left; R, right.

Children with ASD are thought to have undergone impaired early auditory information processing. Some earlier studies have indicated atypical brain response (e.g., P1m) to auditory stimuli in children with ASD [8,9,10]. In fact, P1m is a component of auditory evoked magnetic fields (AEF), which appears in young children 100 ms after stimulation (Figure 1). It has often been investigated because it is the most prominent component, particularly in young children [11,12,13]. In earlier magnetoencephalography (MEG) studies, various names have been assigned to this early prominent component P1m: M50 [8,11,14,15], P1m [16,17], P50m [18], and P100m [12,19]. Their inconsistencies might be explained by the presence of different P1(m) subcomponents in children. Prominence of these subcomponents might depend on the stimulation type, experimental task, or participant age [12]. We have designated this early and most prominent component as P1m. Using human voice stimuli, we have identified the maturational change of P1m in young children in cross-sectional [20,21] and longitudinal design [13,22]. For this study, using pure tone stimuli, we specifically examined the P1m component of the magnetic field response.

Some earlier MEG reports of some studies have described longer P1m latency in young children with ASD [10,15,23]. Roberts and colleagues reported that minimally verbal and nonverbal 8–12-year-old children with ASD showed significantly longer P1m latency compared to children with TD across hemispheres [10]. Another study that included adolescent individuals with ASD (mean age of participants, 11.8 years) indicated that P1m latency in participants without language impairment was shorter than in those with language impairment [8]. Despite mounting evidence indicating a link between P1m latency and each of tone stimuli and language impairment in ASD, no earlier study specifically examined this linkage in ASD patients younger than six years old without language or intellectual disability.

In our earlier study, using the human voice stimulus/ne/, we found greater leftward lateralized P1m intensity in preschool children with TD than in children with ASD [20]. Furthermore, shorter P1m latency in both hemispheres was particularly correlated with higher language-related performance in the children with TD. This correlation was not found for children with ASD. These results of our earlier studies suggest that the early brain auditory system, which processes human voice information, is important for language development in children with but not in children with ASD. Nevertheless, whether brain response to pure tone stimuli is related to language ability in children with TD and children with ASD remains unclear.

This study specifically examines P1m response (latency and intensity) evoked by pure tone stimuli. This study was conducted to investigate whether pure tone evoked P1m is atypical in younger children with ASD (i.e., 5–8 years old) without intellectual disability. Furthermore, based on results of our earlier study, we investigate the relation between brain response to pure tone stimuli and language ability. We hypothesized that children with ASD without intellectual disability show atypical P1m response compared to that of children with TD.

## 2. Results

### 2.1. P1m Latency and Intensity

For children with TD examined for this study, the dipole latencies were 108 ± 10.8 and 97 ± 18.9 ms (mean ± SD), respectively, in the left and right hemispheres. For children with ASD, the dipole latencies were 101 ± 13.0 and 98 ± 20.0 (mean ± SD), respectively, in the left and right hemispheres. Paired *t*-tests revealed significant difference in P1m latency in the left hemisphere between children with TD and children with ASD (ASD, *N* = 26; TD, *N* = 38; t = 2.390, *p* = 0.020) (Table 1). This difference indicates that the children with ASD exhibit significantly shorter P1m latency in the left hemisphere than that exhibited by children with TD. In order to take into account the effect of gender effect on this *t*-test results, the latency of left P1m was examined using ANOVA, including gender factors. As a result, there was a significant difference in the main effect of group (F(1,60) = 7.949, *p* = 0.007), and there was no significant difference in the main effect of gender (F(1,60) = 0.144, *p* > 0.05), no interaction between group and gender was observed.

In the comparison of P1m intensity using repeated two-way ANOVA (subject group × hemisphere), participants in whom P1m was detected only on the left or right side were excluded. Therefore, the number of samples is smaller than for the paired *t*-test. Repeated two-way ANOVA (subject group × hemisphere) revealed a main effect of hemisphere, with results of F(1,29) = 5.890, *p* = 0.022 (ASD, *N* = 18; TD, *N* = 13), indicating shorter P1m responses in the right versus left hemisphere. Effects of the group were not found to be significant (ASD, *N* = 18; TD, *N* = 13; F(1,29) = 3.777; *p* = 0.062). No significant interaction was found between the group and hemisphere. For the children with TD, the dipole intensities were 18.5 ± 6.5 and 13.3 ± 5.0 nAm (mean ± SD), respectively, in the left and right hemispheres. For children with ASD, the dipole intensities were 18.4 ± 7.8 and 15.2 ± 6.8 (mean ± SD), respectively, in the left and right hemispheres. Paired *t*-tests did not show a significant difference in P1m intensity in hemispheres between the two groups. A comparison of P1m intensity using repeated two-way ANOVA (subject group × hemisphere) revealed a main effect of hemisphere, with results of F(1,29) = 7.387, *p* = 0.011 (ASD, *N* = 18; TD, *N* = 13), indicating stronger left than right P1m responses. No significant effect of the group nor of interaction between the group and hemisphere was found. This degree of significance indicates that the P1m intensity in the left hemisphere is significantly higher than in the right hemisphere in both groups.

### 2.2. Correlation between P1m Latency and Language-Related Performances

**Figure 2:**
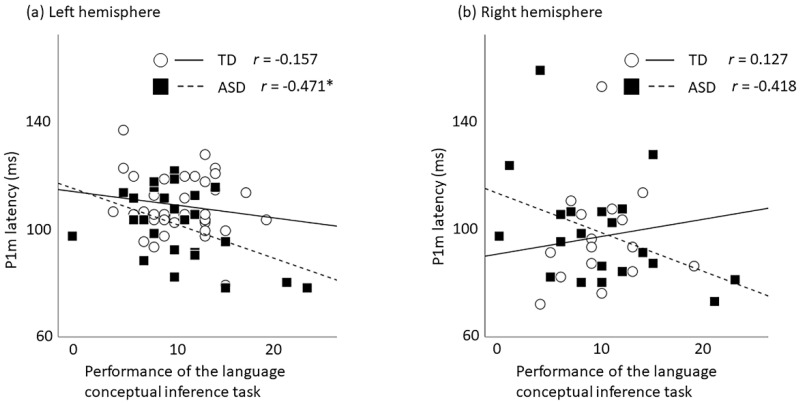
Scatter plot of the P1m latency and the performance of language conceptual inference in both groups. For children with ASD, a shorter P1m latency in the left hemispheres was associated with a higher performance on the language conceptual inference tasks (*p* = 0.015) (**a**). The correlation was not statistically significant in the right hemisphere (*p* = 0.067) (**b**). No significant correlation between the P1m dipole latency and the performance on the language conceptual inference tasks for children with TD: solid line, regression line for children with TD; broken line, regression line for children with ASD. * *p* < 0.025.

Pearson’s correlation coefficients examining the relation between P1m latency (in the right and left hemisphere) and the performance of Japanese adaptation of the Kaufman Assessment Battery for Children (K-ABC) [24] language subtest ‘riddles’ were calculated separately for children with TD and children with ASD. In children with ASD, significant correlation was found between P1m latency in the left hemisphere and the performance of K-ABC language subtest ‘riddles’ (*N* = 26, r = −0.471, *p* = 0.015) (Table 2, Figure 2). No significance was found for P1m latency in the right hemisphere (*N* = 20, r = −0.418, *p* = 0.067). In children with TD, no significant relation between P1m latency and language performance was found in either hemisphere (left, *N* = 38, r = −0.157, *p* = 0.346; right, *N* = 16, r = 0.127, *p* = 0.640).

To examine the effect of age, as a complementary analysis, we performed the multiple regression analysis in which the age of the months and the score of the language subtest ‘riddles’ were included as the independent variables, to examine the relation between P1m latency and the language performance for children with ASD. As a result of multiple regression analysis, in the left hemisphere, the fit of the regression model was not significant (*N* = 26, F(2,23) = 3.308, *p* = 0.055). The language performance ‘riddle’ tended to correlate with the left P1m latency but no significance was found after multiple comparison correction (β = −0.455, *p* = 0.036). In the right hemisphere, the regression model was found to be significant (*N* = 20, F(2,17) = 5.446, *p* = 0.015). Both age in months and the ‘riddle’ language performance were associated significantly with the P1m latency (age in months, β= 0.489, v = 0.025; riddles, β = −0.572, *p* = 0.011).

### 2.3. Correlation between P1m Intensity and Language-Related Performances

Pearson’s correlation coefficients examining the relation between P1m intensity (in the right and left hemisphere) and the performance of K-ABC language subtest ‘riddles’ were calculated separately for children with TD and children with ASD. In both groups, no significant correlation was found between P1m intensity and the performance of language in both hemispheres.

## 3. Discussion

This study investigated AEF evoked by tone stimuli in 5–8-year-old children: some TD and some children with ASD without intellectual disabilities. Of the participants examined for this study, 89% were children aged 5 or 6 years old. This report is the first to describe features of bilateral AEF dipole source in children with ASD without intellectual disabilities who were mainly 5 and 6 years old. A noteworthy finding of this study is that the brain response (P1m) evoked by pure tone in the left hemisphere of children with ASD without intellectual disability was significantly faster than that in the children with TD. Our findings are inconsistent with those of some earlier studies. Matsuzaki et al. [25], Roberts et al. [10,15], Bloy et al. [26], and Williams et al. [27] reported significantly longer P1m latency in children with ASD than in children with TD. However, in those earlier studies, unlike our present study, the participants were older (around 10 years on average). Some participants had language and cognitive delays. Differences in age ranges and cognitive function in children with ASD might give rise to this inconsistency between this study and earlier studies of P1m latency.

For P1m latency, one study has examined children with ASD under six years of age, although their intellectual function is unclear. Stephen et al. [28] examined AEF by tone burst in children with ASD aged 24–62 months. The researchers failed to demonstrate longer early brain response observed in young children with ASD than in normally developing children at around 100 ms after auditory stimulus. Results of some EEG studies also suggest that children with ASD showed shorter latency in a component that appears to correspond to P1m: P1. Donkers et al. [29] reported that children with ASD showed slightly shorter P1 latency, although no significant difference was found between children with ASD and children with TD aged 4–12 years. Orekhova et al. [30] reported that children with ASD without intellectual disabilities aged 3–8 years showed significantly shorter P1 latency to click stimuli than age-matched children with TD. Given that myelination in the white matter enhances nerve conduction velocity considerably and that it engenders shorter latency of the brain response [31,32,33], shorter P1m latency to tone stimuli might reflect the progression of increasing myelination in brain areas related to single sound processing for ASD without intellectual disabilities. In the human central auditory system, early myelin formation is observed in the auditory thalamic cortical axons by 1 year of age, and reaches adult levels around 4–5 years of age [34,35]. Since the latency of the tone-evoked response has been reported to be shorten with the maturation of such white matter (i.e., myelination) [36], the latency of the tone-evoked response reflects the degree of maturation of the auditory pathway. Therefore, the difference in the latency of P1m between TD and ASD observed in this study may reflect an atypical brain maturation process of the central auditory pathway in the early (infant) stage children with ASD. Findings about atypical brain maturation during young childhood may contribute to as an early objective physiological indicator of ASD without intellectual disabilities and as verification of early intervention effects. Furthermore, it is meaningful to develop the physiological index specialized for language evaluation, as it is one of the major diversities of ASD. One of the promising strategies to develop effective intervention for each child is to evaluate the language and develop the corresponding physiological indices, which will contribute to precision medicine in the future.

Recent reports of imaging studies have described aberrant development of white matter [37] and atypical brain developmental trajectory [38,39] in early stage ASD, which interferes with information processing from the external world and which might cause symptoms that are characteristic of ASD. Recent fMRI studies specifically emphasizing functional connectivity provide evidence for widespread hyper-connectivity in children with ASD (7–12 y.o.) in contrast to the hypo-connectivity observed in adolescents and adults with ASD [39]. Our result (i.e., shorter P1m latency in young children with ASD) indicates that, in young children with ASD without intellectual delay, white matter maturation might be accelerated, which results in neural hyper-connectivity in early childhood.

Regarding the relation between auditory brain response and language ability, pure tone evoked P1m is not related to language ability in children with TD, although our earlier studies have demonstrated that human-voice-induced P1m is associated with language development in children with TD. Although no definitive conclusions can be drawn from our earlier and current studies because of differences in subject age, the results of this study underscore the necessity of maturation in the left auditory cortex to human voice for language development in children with TD but not in children with ASD. In the present study, children with ASD but not children with TD demonstrated a relation between tone-evoked P1m latency and language ability, whereas our earlier studies failed to demonstrate that human-voice-induced P1m is associated with language development in children with ASD. These results from our earlier studies and current results suggest the necessity of maturation in the auditory cortex to pure tone processing for language development in children with ASD, but not in children with TD. This dissociation for these correlations (i.e., maturation of auditory system and language ability) among stimulus features suggests different neural bases involved in language acquisition for TD and for ASD.

Our results suggest that overmaturation in white matter during this early period is associated with language development in ASD without intellectual disabilities. Myelination of language-related areas in the human brain progresses rapidly from birth to three years of age. The respective volumes of myelinated white matter in language-related temporal and frontal regions and in the central sensorimotor region are reportedly associated with children’s vocabulary acquisition [40]. Presumably, maturation of the white matter of the auditory cortex, which corresponds to the processing of a simple sound, might be more important for language development in children with ASD. Results of this study underscore the recognition that maturation of the auditory cortex for a simple sound tone, rather than the human voice, can be a biomarker for language development in children with ASD. Some studies using functional connectivity analysis across the whole brain showed widespread hyper-connectivity [39] alongside hypo-connectivity in voice-processing areas [41] and both hypo-connectivity and hyper-connectivity between subsets of specific regions [42]. This study examined brain cortical processing for only a single auditory stimulus. Therefore, it cannot be concluded whether it reflects hypermaturation of the global level across the whole brain during this childhood period or hyperconnectivity between specific regions (i.e., auditory processing).

The ‘riddle’ language test, results of which were associated with the P1m latency in children with ASD, is a task that requires language listening comprehension skill and expressive language skills [24]. Language comprehension and production are thought to involve a network of frontal, parietal, and temporal lobes interconnected by two major white matter pathways in the left hemisphere [43,44,45,46], Our results suggest that in children with ASD aged 5–8 years with no intellectual disability, compared to children with TD, the brain network that processes pure sound stimuli accounts for a larger proportion of the extensive brain network for language acquisition described above. In addition, the results of this study suggest that the development of language processing networks in young children with ASD differs from that of children with typical development.

The present study has some limitations. First, we investigated AEFs with a short ISI (1200 ms); in younger children, the duration of this ISI was insufficient to allow a return to baseline from a stimulus. Although long (i.e., more than 2 s) ISIs would enhance the amplitude of auditory evoked responses and improve the signal-to-noise ratio, we used only short ISI (1200 ms) in this study. Therefore, P1m components in the present study may have been influenced by the neuronal refractory period for stimulation. Second, previous reports have indicated that in addition to alterations in cortical auditory processes, the ASD population exhibits increased rates of brainstem or peripheral hearing dysfunction [47]. Dysfunctions in both peripheral and central auditory processing also distort the cortical AEF components. Therefore, further studies—including analyses of brainstem function and fine peripheral hearing function—are necessary to draw definitive conclusions. Third, we eliminated any contaminated MEG data, such as data obtained when clear head movement occurred. However, differences in the fine head movements of children with ASD and children with TD may have confounded the study results.

## 4. Materials and Methods

### 4.1. Participants

The clinical group included 29 children with ASD (8 girls, 21 boys) of 60–98 months of age, mean ± SD = 74.7 ± 10.8 months. They were recruited from Kanazawa University and prefectural hospitals in the Kanazawa or Toyama area. The ASD diagnosis was made by a psychiatrist and a clinical speech therapist. The speech therapist, who was well trained and who has an Autism Diagnostic Observational Schedule research license as well as more than 10 years of experience in ASD treatment, used the Autism Diagnostic Observational Schedule, Generic (ADOS-G) [48] or Autism Diagnostic Observational Schedule-2 [49]. The definitive diagnosis of ASD was made by a psychiatrist with more than 10 years of experience in ASD using the Diagnostic Interview for Social and Communication Disorders (DISCO) [50], and the DSM-IV criteria [51] or the DSM-V [1] at the time of the MEG and the Kaufman Assessment Battery for Children (K-ABC) [24] data acquisition. These 26 children with ASD satisfied the diagnosis of childhood autism (*n* = 20) or atypical autism (*n* = 6) using the DISCO. Children who were below the DISCO cutoff levels were included in this study if they met the criteria for ASD using both the DSM-IV criteria and ADOS (i.e., 3 children). As controls, we examined 46 children with TD (5 girls, 41 boys), 63–91 months old, mean ± SD = 70.3 ± 5.9 months, with no reported behavioral or language problems. The children with TD were matched to ASD subjects according to age in months. All TD subjects were native Japanese children with no prior or existing developmental, learning, or behavioral problems according to information obtained from a questionnaire completed by their parents. All participants had normal hearing abilities according to available medical records. Left-hand or right-hand dominance was determined based on the Edinburgh handedness inventory [52]. The following results were obtained: children with TD (right = 42, left = 4) and children with ASD (right = 27, left = 2). Physical data for all participants are presented in Table 3. All children participated in cognitive tasks and MEG measurements. The parents agreed to the participation of their children in the study with full knowledge of the experiment characteristics of the research. Their written informed consent was obtained before the study. The Ethics Committee of Kanazawa University Hospital approved the methods and procedures 2016-430 (759)(18/01/2012), all of which were performed in accordance with the Declaration of Helsinki.

### 4.2. Cognitive and Language Performance Measurements

Cognitive function was evaluated from Japanese adaptation of the Kaufman Assessment Battery for Children (K-ABC), which is typically used to assess the cognitive skills of children aged 30–155 months [24]. To confirm the standardized score of the mental processing scales in children, subtests that were complementary to the age (in months) of the children were used in this battery. Inclusion criteria for all participants included 70 to 130 scores on the K-ABC mental processing scale. Because we have demonstrated significant correlation between the early component of the voice-evoked AEF (i.e., P1m latency) and the performance of a language-related task (i.e., a subtest of K-ABC ‘riddles’) in children with TD in our earlier study [53], to confirm the relation between the P1m latency evoked by non-voice stimuli and language ability, we used the ‘riddles’ subtest for this study. The riddle task consists of 32 questions, which are presented in ascending order of difficulty. The linguistic level is defined by the child’s degree of achievement. The K-ABC ‘riddles’ subtest reflects conceptual language inference abilities [24].

### 4.3. Auditory Stimuli

Stimuli were sinusoidal tones. For this study, we used typical oddball sequences consisting of standard stimuli (523.3 Hz, 400 times, 75%) and deviant stimuli (1046.6 Hz, 100 times, 25%). We adopted only the standard stimuli for subsequent equivalent current dipole (ECD) estimations. The stimulus duration was 50 ms. Auditory stimuli were presented using presentation software (Neurobehavioral Systems Inc., Berkeley, CA, USA). The interstimulus interval (ISI) was 1200 ms. The stimulus had an intensity level of approximately 71 dB (A-weighted) at the head position against a background noise level of 43 dB. Intensity was measured using an integrating sound level meter (LY20; Yokogawa Analytical Systems Inc., Tokyo, Japan). The stimulus was presented to the participants binaurally through a panel speaker (AA-160e; Panphonics Oy., Tampere, Finland) to the dewar. The recording was approximately 10 min long.

### 4.4. Magnetoencephalography Recording

The MEG data were acquired using a whole-head 151 channel child-customized MEG system (PQ 1151R; Yokogawa/KIT, Kanazawa, Japan) in a magnetically shielded room (Daido Steel Co., Ltd., Nagoya, Japan) installed at the MEG Center of Ricoh Co., Ltd. (Tokyo, Japan). The MEG signals were digitized at 2000 Hz and low-pass filtered at 200 Hz. We instructed participants not to move their head or body too much during the experiments to avoid contamination and artifacts. One research team member remained in a shielded room to encourage the child to maintain a steady body position when necessary. Simultaneously, the participants were monitored carefully using a video monitoring system. Stimuli were presented while the children lay in the supine position on the bed and viewed silent video programs projected onto a screen. The head location relative to the MEG device helmet was measured using four coils attached on the head surface as fiduciary points with respect to the landmarks (bilateral preauricular points, Cz and 5 cm anterior part from Cz) for children. Before the MEG session, a three-dimensional digitizer (FASTRAK; Polhemus, Colchester, VT, USA) was used to digitize the head surface points and fiduciary landmarks of the participant. After MEG recording, the positioning coils were replaced with MRI-visible markers.

Brain structural images were obtained individually for source reconstruction from participants except two children with ASD using a 1.5 T MRI scanner (SIGNA Explorer; GE Healthcare, Chicago, IL, USA). For each participant, an MRI scan was acquired using the T1-weighted gradient echo and Silenz pulse sequence (TR = 435.68 ms, TE = 0.024 ms, flip angle = 7°, FOV = 220 mm, matrix size = 256 × 256 pixels, slice thickness = 1.7 mm, and 130 transaxial images). A spherical model was used for ECD analysis for two children who failed to complete MRI imaging [54].

### 4.5. Data Analysis

Epochs of 150 ms pre-stimulus to 800 ms post-stimulus were defined from continuous recording. Subsequent segments (at least 150 for standard stimuli) were averaged for each of the sensors after baseline correction (−50 to 0 ms). The segments that were contaminated with artifacts (i.e., eye-blink, and eye and body movements which are typically more than ±4 pT) were excluded from analyses. A single ECD model was used to estimate the current sources in the activated cerebral cortex using more than 30 sensors for each hemisphere (left and right). To estimate localization of the current sources, a MegLaboratory 160 (Yokogawa/KIT, Kanazawa, Japan) was used.

To identify the P1m component, we accepted estimated ECDs when (1) the goodness of fit (GOF) exceeded 80%, (2) the location of the estimated dipoles using a single ECD model was stabilized within ±5 mm of each coordinate for at least 6 ms during the target response period, (3) the dipole intensities were ≤80 nAm, and (4) the direction of the estimated ECD was in an anterosuperior orientation. The latency time point was defined as the maximum estimated dipole intensity value obtained in accordance with the above criteria within a time window of 70–150 ms.

### 4.6. Statistical Analysis

We used software (SPSS ver. 20.0; IBM Corp., Armonk, NY, USA) for statistical analyses. To evaluate differences of dipole intensity and latency of the P1m component between the TD and children with ASD, we applied two-sample *t*-tests (two-tailed) to data of each hemisphere. The alpha level was adjusted to 0.05/2 = 0.025 for statistical analysis for each hemisphere. Furthermore, to test the left–right interaction, we performed repeated two-way ANOVA for P1m dipole latency and intensity. The between-subject variable was the group (TD vs. ASD). The within-subject variable was the hemisphere (left and right). Statistical significance was inferred for *p* < 0.05.

To evaluate correlation between the P1m component (i.e., the intensity and latency) and the language-related score (i.e., the performance of ‘riddles’), Pearson’s correlation coefficient was used. Analyses were conducted separately for the right and left hemispheres. The alpha level was adjusted to 0.05/2 = 0.025 for statistical analysis. Furthermore, we used a multiple regression analysis to assess the effects of independent variables. As a between-person covariate, age in months and the language score were included in the regression model. Statistical significance was defined as *p* < 0.05/2 = 0.025.

## Figures and Tables

**Table 1:** P1m component.

	TD	ASD	t	*p*
Latency (ms)				
Left (N)	38	26		
	108 (10.8)	101 (13.0)	2.390	0.020 *
Right (N)	16	20		
	97 (18.9)	98 (20.0)	−0.232	0.818
Intensity (nAm)				
Left (N)	38	26		
	18.5 (6.5)	18.4 (7.8)	0.059	0.953
Right (N)	16	20		
	13.3 (5.0)	15.2 (6.8)	−0.966	0.341

* *p* < 0.025.

**Table 2:** Correlation between P1m latency/intensity and language performance.

	TD		ASD	
	r	*p*	r	*p*
Latency				
Left	−0.157	0.346	−0.471	0.015 *
Right	0.127	0.640	−0.418	0.067
Intensity				
Left	0.205	0.217	−0.102	0.619
Right	0.523	0.038	−0.333	0.151

* *p* < 0.025.

**Table 3:** Clinical characteristics.

	TD	ASD	t	*p*
Number of participants	46	29		
Gender (male/female)	41/5	21/8		n.s.
Chronological age (months)	70.3 (63–91)	74.7 (60–98)		n.s.
Handedness (right/left)	42/4	27/2		n.s.
K-ABC ^1^–MPS ^2^	104.7 (12.9)	91.4 (11.7)	4.488	<0.001
K-ABC ^1^–AS ^3^	101.4 (13.9)	95.2 (13.4)	1.913	n.s.

^1^ K-ABC, Kaufman Assessment Battery for Children. ^2^ MPS, Mental Processing Scale. ^3^ AS, Achievement Scale. The values are the mean (standard deviation) for chronological age, head size, scales on the K-ABC. n.s., no significance.

## Data Availability

The datasets collected during or analyzed during the current study are available from the corresponding author upon reasonable request.

## References

[1] American Psychiatric Association (2013). Diagnostic and Statistical Manual of Mental Disorders.

[2] Osterling J., Dawson G. (1994). Early recognition of children with autism: A study of first birthday home videotapes. J. Autism Dev. Disord..

[3] Carper R.A., Moses P., Tigue Z.D., Courchesne E. (2002). Cerebral lobes in autism: Early hyperplasia and abnormal age effects. Neuroimage.

[4] Schumann C.M., Bloss C.S., Barnes C.C., Wideman G.M., Carper R.A., Akshoomoff N., Pierce K., Hagler D., Schork N., Lord C. (2010). Longitudinal magnetic resonance imaging study of cortical development through early childhood in autism. J. Neurosci..

[5] Shen M.D., Nordahl C.W., Young G.S., Wootton-Gorges S.L., Lee A., Liston S.E., Harrington K.R., Ozonoff S., Amaral D.G. (2013). Early brain enlargement and elevated extra-axial fluid in infants who develop autism spectrum disorder. Brain.

[6] Hazlett H.C., Gu H., Munsell B.C., Kim S.H., Styner M., Wolff J.J., Elison J.T., Swanson M.R., Zhu H., Botteron K.N. (2017). Early brain development in infants at high risk for autism spectrum disorder. Nature.

[7] Piven J., Elison J.T., Zylka M.J. (2017). Toward a conceptual framework for early brain and behavior development in autism. Mol. Psychiatry.

[8] Cardy J.E.O., Flagg E.J., Roberts W., Roberts T.P. (2008). Auditory evoked fields predict language ability and impairment in children. Int. J. Psychophysiol..

[9] Matsuzaki J., Kagitani-Shimono K., Goto T., Sanefuji W., Yamamoto T., Sakai S., Uchida H., Hirata M., Mohri I., Yorifuji S. (2012). Differential responses of primary auditory cortex in autistic spectrum disorder with auditory hypersensitivity. Neuroreport.

[10] Roberts T.P.L., Matsuzaki J., Blaskey L., Bloy L., Edgar J.C., Kim M., Ku M., Kuschner E.S., Embick D. (2019). Delayed M50/M100 evoked response component latency in minimally verbal/nonverbal children who have autism spectrum disorder. Mol. Autism.

[11] Cardy J.E.O., Ferrari P., Flagg E.J., Roberts W., Roberts T.P.L. (2004). Prominence of M50 auditory evoked response over M100 in childhood and autism. Neuroreport.

[12] Orekhova E.V., Butorina A.V., Tsetlin M.M., Novikova S.I., Sokolov P.A., Elam M., Stroganova T.A. (2012). Auditory magnetic response to clicks in children and adults: Its components, hemispheric lateralization and repetition suppression effect. Brain Topogr..

[13] Yoshimura Y., Hasegawa C., Ikeda T., Saito D.N., Hiraishi H., Takahashi T., Kumazaki H., Kikuchi M. (2020). The maturation of the P1m component in response to voice from infancy to 3 years of age: A longitudinal study in young children. Brain Behav..

[14] Oram Cardy J.E., Flagg E.J., Roberts W., Brian J., Roberts T.P. (2005). Magnetoencephalography identifies rapid temporal processing deficit in autism and language impairment. Neuroreport.

[15] Roberts T.P.L., Khan S.Y., Rey M., Monroe J.F., Cannon K., Blaskey L., Woldoff S., Qasmieh S., Gandal M., Schmidt G.L. (2010). MEG detection of delayed auditory evoked responses in autism spectrum disorders: Towards an imaging biomarker for autism. Autism Res..

[16] Pihko E., Mickos A., Kujala T., Pihlgren A., Westman M., Alku P., Byring R., Korkman M. (2006). Group intervention changes brain activity in bilingual language-impaired children. Cereb. Cortex.

[17] Pihko E., Kujala T., Mickos A., Alku P., Byring R., Korkman M. (2008). Language impairment is reflected in auditory evoked fields. Int. J. Psychophysiol..

[18] Tavabi K., Obleser J., Dobel C., Pantev C. (2007). Auditory evoked fields differentially encode speech features: An MEG investigation of the P50m and N100m time courses during syllable processing. Eur. J. Neurosci..

[19] Orekhova E.V., Tsetlin M.M., Butorina A.V., Novikova S.I., Gratchev V.V., Sokolov P.A., Elam M., Stroganova T.A. (2012). Auditory cortex responses to clicks and sensory modulation difficulties in children with Autism Spectrum Disorders (ASD). PLoS ONE.

[20] Yoshimura Y., Kikuchi M., Shitamichi K., Ueno S., Munesue T., Ono Y., Tsubokawa T., Haruta Y., Oi M., Niida Y. (2013). Atypical brain lateralisation in the auditory cortex and language performance in 3- to 7-year-old children with high-functioning autism spectrum disorder: A child-customised mag-netoencephalography (MEG) study. Mol. Autism.

[21] Yoshimura Y., Kikuchi M., Hiraishi H., Hasegawa C., Takahashi T., Remijn G.B., Oi M., Munesue T., Higashida H., Minabe Y. (2016). Synchrony of auditory brain responses predicts behavioral ability to keep still in children with autism spectrum disorder: Auditory-evoked response in children with autism spectrum disorder. NeuroImage Clin..

[22] Yoshimura Y., Kikuchi M., Ueno S., Shitamichi K., Remijn G.B., Hiraishi H., Hasegawa C., Furutani N., Oi M., Munesue T. (2014). A longitudinal study of auditory evoked field and language development in young children. Neuroimage.

[23] Edgar J.C., Lanza M.R., Daina A.B., Monroe J.F., Khan S.Y., Blaskey L., Cannon K.M., Jenkins J., Qasmieh S., Levy S.E. (2014). Missing and delayed auditory responses in young and older children with autism spectrum disorders. Front. Hum. Neurosci..

[24] Kaufman A., Kaufman N. (1983). Kaufman Assessment Battery for Children.

[25] Matsuzaki J., Ku M., DiPiero M., Chiang T., Saby J., Blaskey L., Kuschner E.S., Kim M., Berman J.I., Bloy L. (2019). Delayed Auditory Evoked Responses in Autism Spectrum Disorder across the Life Span. Dev. Neurosci..

[26] Bloy L., Ku M., Edgar J.C., Miller J.S., Blaskey L., Ross J., Roberts T.P. (2019). Auditory evoked response delays in children with 47,XYY syndrome. Neuroreport.

[27] Williams Z.J., Abdelmessih P.G., Key A.P., Woynaroski T.G. (2020). Cortical auditory processing of simple stimuli is altered in autism: A meta-analysis of auditory evoked responses. Biol. Psychiatry Cogn. Neurosci. Neuroimaging.

[28] Stephen J.M., Hill D.E., Peters A., Flynn L., Zhang T., Okada Y. (2017). Development of auditory evoked responses in normally developing preschool children and children with autism spectrum disorder. Dev. Neurosci..

[29] Donkers F.C.L., Schipul S.E., Baranek G.T., Cleary K.M., Willoughby M.T., Evans A.M., Bulluck J.C., Lovmo J.E., Belger A. (2015). Attenuated Auditory event-related potentials and associations with atypical sensory response patterns in children with autism. J. Autism Dev. Disord..

[30] Orekhova E.V., Stroganova T.A., Prokofyev A.O., Nygren G., Gillberg C., Elam M. (2008). Sensory gating in young children with autism: Relation to age, IQ, and EEG gamma oscillations. Neurosci. Lett..

[31] Roncagliolo M., Benítez J., Eguibar J.R. (2000). Progressive deterioration of central components of auditory brainstem responses during postnatal development of the myelin mutant taiep rat. Audiol. Neurotol..

[32] Lee D.L., Strathmann F.G., Gelein R., Walton J., Mayer-Proschel M. (2012). Iron deficiency disrupts axon maturation of the developing auditory nerve. J. Neurosci..

[33] Daneshvarfard F., Abrishami Moghaddam H., Kongolo G., Wallois F., Mahmoudzadeh M. (2020). Functional and structural cor-relates of the preterm infant’s brain: Relating developmental changes of auditory evoked responses to structural maturation. Brain Struct. Funct..

[34] Yakovlev P.I., Lecours A.R., Minkocoaki A. (1967). The Myelogenetic Cycles of Regional Maturation of the Brain. Regional Development of Brain Early Life.

[35] Kinney H.C., Brody B.A., Kloman A.S., Gilles F.H. (1988). Sequence of central nervous system myelination in human infancy. II. Patterns of myelination in autopsied Infants. J. Neuropathol. Exp. Neurol..

[36] Roberts T.P.L., Khan S.Y., Blaskey L., Dell J., Levy S.E., Zarnow D.M., Edgar J.C. (2009). Developmental correlation of diffusion anisotropy with auditory-evoked response. Neuroreport.

[37] Wolff J.J., Gu H., Gerig G., Elison J.T., Styner M., Gouttard S., Botteron K.N., Dager S.R., Dawson G., Estes A.M. (2012). Differences in white matter fiber tract development present from 6 to 24 months in infants with autism. Am. J. Psychiatry.

[38] Redcay E., Courchesne E. (2005). When is the brain enlarged in autism? A meta-analysis of all brain size reports. Biol. Psychiatry.

[39] Uddin L.Q., Supekar K., Menon V. (2013). Reconceptualizing functional brain connectivity in autism from a developmental perspective. Front. Hum. Neurosci..

[40] Pujol J., Soriano-Mas C., Ortiz H., Sebastian-Galles N., Losilla J.M., Deus J. (2006). Myelination of language-related areas in the developing brain. Neurology.

[41] Abrams D.A., Lynch C.J., Cheng K.M., Phillips J., Supekar K., Ryali S., Uddin L.Q., Menon V. (2013). Underconnectivity between voice-selective cortex and reward circuitry in children with autism. Proc. Natl. Acad. Sci. USA.

[42] Lynch C.J., Uddin L.Q., Supekar K., Khouzam A., Phillips J., Menon V. (2013). Default mode network in childhood autism: Posteromedial cortex heterogeneity and relationship with social deficits. Biol. Psychiatry.

[43] Friederici A.D., Rüschemeyer S.-A., Hahne A., Fiebach C.J. (2003). The Role of Left Inferior Frontal and Superior Temporal Cortex in Sentence Comprehension: Localizing Syntactic and Semantic Processes. Cereb. Cortex.

[44] Hickok G., Poeppel D. (2004). Dorsal and ventral streams: A framework for understanding aspects of the functional anatomy of language. Cognition.

[45] Catani M., Jones D.K., Ffytche D.H. (2005). Perisylvian language networks of the human brain. Ann. Neurol..

[46] Rolheiser T., Stamatakis E.A., Tyler L.K. (2011). Dynamic processing in the human language system: Synergy between the arcuate fascicle and extreme capsule. J. Neurosci..

[47] Demopoulos C., Lewine J.D. (2016). audiometric profiles in autism spectrum disorders: Does Subclinical hearing loss impact communication?. Autism Res..

[48] Lord C., Risi S., Lambrecht L., Cook E.H., Leventhal B.L., DiLavore P.C., Pickles A., Rutter M. (2000). The autism diagnostic observation schedule-generic: A standard measure of social and communication deficits associated with the spectrum of autism. J. Autism Dev. Disord..

[49] Lord C., Rutter M., DiLavore P.C. (2012). Autism Diagnostic Observation Schedule.

[50] Wing L., Leekam S.R., Libby S.J., Gould J., Larcombe M. (2002). The diagnostic interview for social and communication disorders: Background, inter-rater reliability and clinical use. J. Child Psychol. Psychiatry.

[51] American Psychiatric Association (1994). Diagnostic and Statistical Manual of Mental Disorders.

[52] Oldfield R. (1971). The assessment and analysis of handedness: The Edinburgh inventory. Neuropsychology.

[53] Yoshimura Y., Kikuchi M., Ueno S., Okumura E., Hiraishi H., Hasegawa C., Remijn G.B., Shitamichi K., Munesue T., Tsubokawa T. (2013). The brain’s response to the human voice depends on the incidence of autistic traits in the general population. PLoS ONE.

[54] Yoshimura Y., Kikuchi M., Shitamichi K., Ueno S., Remijn G.B., Haruta Y., Oi M., Munesue T., Tsubokawa T., Higashida H. (2012). Language performance and auditory evoked fields in 2- to 5-year-old children. Eur. J. Neurosci..

